# First Case of Macrocephaly, Dysmorphic Facies, and Psychomotor Retardation Harboring Co-inherited Variants in HERC1 and PMP22 Genes from Iran: Two Novel Variants

**DOI:** 10.34172/aim.31593

**Published:** 2024-12-01

**Authors:** Azadeh Reshadmanesh, Shima Dehdahsi, Fatemeh Ahangari, Kimia Kahrizi, Ariana Kariminejad, Shokouh Sadat Mahdavi, Saeed Talebi, Hossein Najmabadi

**Affiliations:** ^1^Genetics Research Center, University of Social Welfare and Rehabilitation Sciences, Tehran, Iran; ^2^Kariminejad-Najmabadi Pathology & Genetics Center, Tehran, Iran; ^3^Genetic Clinic of Tehran Welfare Organization, Tehran, Iran; ^4^Department of Medical Genetics, School of Medicine, Iran University of Medical Sciences, Tehran, Iran

**Keywords:** Charcot–Marie–Tooth diseases, Concomitant variants, *HERC1*, *PMP22* duplication, Whole exome sequencing

## Abstract

Here, we report a case with concomitant variants: a novel homozygous *HERC1* gene variant and a novel heterozygous *PMP22* duplication. The 2-year-old male presented with seizures, developmental delay, macrocephaly, hypotonia, unilateral hypertrophy, thoracic scoliosis, normal brain MRI, and elevated homocysteine level which normalized after treatment. Whole exome sequencing (WES) revealed a co-occurrence of a homozygous novel likely pathogenic variant in the *HERC1* gene (NM_003922.3:c.1280dup (p.ILe469Aspfs*33) and a novel heterozygous large duplication of exon 1-5 in the *PMP22* gene, which has not been reported previously. The case underscores the challenges in understanding genotype-phenotype correlations and suggests a potential interplay between these genetic variants in shaping the current and future clinical phenotype of the patient. In the case of genetic diseases, this event may have important implications on family members’ counseling, and concomitant variants in Charcot–Marie–Tooth (CMT) families should be considered when significant intra-familial clinical heterogeneity is observed.

## Introduction

 Identification of genetic variants contributing to complex pediatric phenotypes is challenging. HERC1 is a substantial protein involved in cellular processes like intracellular membrane trafficking and specific target ubiquitination which plays a significant role in eukaryotic cell function.^1^ It is presumed to regulate the mTOR pathway through its interaction with the TSC1–TSC2 complex.^2^

 Pathogenic variants in this gene contributes to macrocephaly, dysmorphic facies, and psychomotor retardation (MDFPMR: OMIM #617011), an autosomal recessive neurodevelopmental disorder characterized by oversize head and somatic overgrowth at birth and global developmental delay, often exhibiting dysmorphic facial features and persistent macrocephaly, with some normalization of increased birth weight with age. Patients may manifest other neurologic features, including hypotonia, seizures, and gait ataxia, accompanied by severe intellectual impairment.^3^

 On the other hand, hereditary motor and sensory neuropathy, known as Charcot–Marie–Tooth (CMT) disease, arises from mutations in various genes.^4,5^ Until now, over 100 genes have been identified with a causative role in CMT disease (http://neuromuscular.wustl.edu/time/hmsn.html), among which peripheral myelin protein 22 (*PMP22*) is the most common causative gene for CMT1, primarily responsible for myelin adhesion and maintenance.^6^ Duplication in the *PMP22* gene leads to CMT type 1A (CMT1A), presenting reduced nerve conduction velocity, usually evident in the first decade of life.

 Here, we present a 2-year-old male exhibiting seizures, developmental delay, macrocephaly, hypotonia, unilateral hypertrophy, and thoracic scoliosis. Despite extensive clinical evaluation, the precise etiology remained elusive until genomic analysis revealed a homozygous novel likely pathogenic variant in the *HERC1* gene, along with a heterozygous duplication in the *PMP22* gene. Such a rare concomitant variants sheds light on potential genetic determinants contributing to this intricate clinical manifestation. Notably, detection of the *PMP22* duplication underscores the necessity of continued clinical monitoring in this case, given that CMT1A symptoms typically emerge after the age of two.

## Case Report

**Figure 1:**
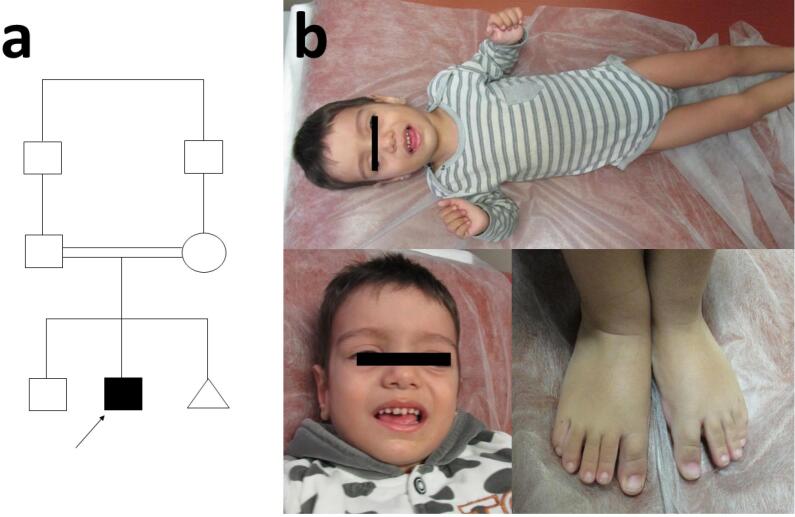
(a) Pedigree of the Patient, (b) The Physical Characteristics of the Patient. Note the large head, prominent forehead, long face, large and low set ears, and elongated limbs, apparent in many of the affected individuals

 The proband was a two-year-old boy resulting from a consanguineous marriage (Figure 1), who referred to Kariminejad-Najmabadi Pathology and Genetic Center, Tehran, Iran, due to delayed neurodevelopment and hypotonia. Delivery was via cesarean section (due to a previous Cesarean-section), at 38 weeks of gestation, had a birth weight of 4 kg (percentile: 89.6%), head circumference of 35 cm (percentile: 66.3%), and length of 52 cm (percentile: 86.9%). During the prenatal period, in the fourth month of pregnancy, fetal ultrasound revealed the presence of unilateral choroid plexus cyst and increased echogenicity of the intestines. Evaluation of the amniocentesis sample for chromosomal anomalies, utilizing both karyotyping and QF-PCR, was found to be normal. Unilateral hydronephrosis was reported in the seventh month of pregnancy. In the neonatal period, due to episodes of lethargy on the 10th day after birth, the infant was hospitalized for 10 days with a diagnosis of urinary tract infection. From two months onwards, the infant exhibited overgrowth. Regression of development occurred following vaccination at two months. The child had a coarse facial appearance during examination. Growth parameters, especially the head circumference, were above normal (macrocephaly). Mild hypertrophy was observed on the right side of the body. The child had generalized hypotonia, speech disorder, and cognitive impairment. Metabolic evaluation was conducted suspecting inherited metabolic diseases. In the metabolic evaluation, the levels of plasma lactate and ammonia were within the normal range. Homocysteine levels were normal in one instance and slightly elevated in another (39.9 µmol/L, reference range: 7.9-16.3 µmol/L ), exceeding the normal limit. There was also a mild and nonspecific increase in urinary methylmalonic acid. Blood gas analysis also indicated mild metabolic acidosis.

 Blood samples were taken from the proband, the parents, and the unaffected sibling. DNA samples were extracted from the proband’s blood using the salting-out method and whole exome sequencing (WES) was performed for the proband. Enrichment was carried out using Twist Exomes V2 (Twist Bioscience, South San Francisco, CA, USA) following the manufacturer’s protocol and WES was performed by the Illumina NovaSeq 6000 platform (Illumina, San Diego, California, USA), to obtain paired-end sequencing Fastq files. This was followed by quality control analysis using the FastQC toolkit, and alignment to the human reference genome build GRCh37 (hg19) through the Illumina Dragen BioIT Platform. The Illumina Dragen haplotype variant calling system was then applied to identify single nucleotide alterations, as well as small insertions and deletions. Finally, the annotation of the identified SNV and small indel variants was outputted by VarSeq^TM^ V2 software (Golden Helix, Inc., Bozeman, MT, https://www.goldenhelix.com/). Filtering the common variants using the latest versions of population databases such as 1000G (http://www.1000genomes.org), the Exome Variant Server (http://evs.gs.washington.edu), EXAC (http://exac.broadinstitute.org), gnomAD (https://gnomad.broadinstitute.org), Iranome (http://www.iranome.ir), and internal databases, interpretation, and reporting of SNV and small indel variants were performed as previously described.^7^ For the analysis of copy number variations (CNVs), the Illumina Dragen platform was employed, utilizing its genome-wide depth-based CNV caller. Annotation of CNVs was performed using AnnotSV, an integrated online tool for structural variations annotation.^8^ For filtering common CNVs, gnomAD, DGV (Database of Genomic Variants) (http://dgv.tcag.ca), and dbVar (https://www.ncbi.nlm.nih.gov/dbvar/) were employed. The final candidate variants (see Supplementary data) were classified following the American College of Medical Genetics and Genomics/Association for Molecular Pathology (ACMG/AMP) guidelines and in accordance with the Clinical Genome (ClinGen) recommendations for utilizing the ACMG/AMP criteria.

 Confirmation of the detected variant as well as segregation study in the family was performed with the help of conventional Sanger sequencing, resulting in identification of a novel homozygous frameshift variant in the *HERC1* gene (NM_003922.3:c.1280dup (p.ILe469Aspfs*33)) which met these criteria under a recessive inheritance model. The c.12804dup variant in the *HERC1* gene has not been published as a mutation, nor has it been documented as a benign polymorphism to date. This genetic alteration results from a duplication of one nucleotide at position 12804, inducing a translational frameshift that predicts an alternate stop codon after 33 amino acids. This change is expected to result in loss of function through either premature protein truncation or nonsense-mediated mRNA decay. This particular variant is not present in the NHLBI Exome Variant Server, the Genome Aggregation Database (gnomAD) or Iranome. In concordance, the *in-silico* prediction program (MutationTaster) supports the probable pathogenicity of this variant. Consequently, based on the currently available information and adhering to the ACMG guideline, this variant is classified as a likely pathogenic variant.

 WES-based CNV analysis for this individual revealed the heterozygous exon 1-5 duplication in *PMP22* gene defined as NC_000017.11:g.(?_14095305-15477497_?)dup. This variant constitutes a gross duplication of the genomic region encompassing full coding sequence of the *PMP22* gene (NM_000521.4). Although this specific large duplication has not previously been documented, duplications of chromosome 17p11.2 region which contain the *PMP22* gene causes Charcot-Marie-Tooth type 1A (CMT1A) and have been shown to lead to increased gene dosage as the functional defect in patients with CMT1A.^9^ Based on these considerations, this variant was classified as pathogenic. Confirmation of the detected *PMP22* duplication was performed by the multiplex ligation probe amplification (MLPA) technique. This important finding of the concomitant occurrence of a loss-of-function mutation in *HERC1* and a *PMP22* duplication carries substantial significance, marking a novel observation absent from the current body of literature. This also emphasizes the significance of CNV analysis, and highlights its crucial role alongside single nucleotide variant (SNV) and small insertion-deletion (indel) investigations in unraveling the intricate genetic landscape underlying complex pediatric cases, especially those with neurodevelopmental presentations, for early interventions.

## Discussion

 We identified a novel loss-of-function homozygous variant (NM_003922.3:c.1280dup) in *HERC1* in a proband with a spectrum of neurological and musculoskeletal manifestations including developmental delay, macrocephaly, and neurological abnormalities. Disorders caused by *HERC1* variants are presumably rare.

**Figure 2:**
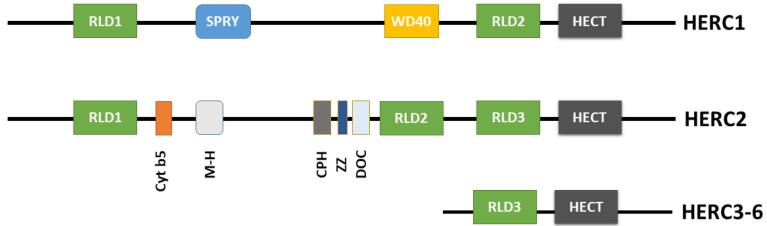
HERC Family Structural Domains Schematic Representation: RLD (RCC1 (regulator of chromosome condensation 1)-like domain), SPRY (spl A and RyR), BH3 (Bcl-2 homology domain 3), WD40 (G protein β-subunit like repeats), HECT (homologous to the E6AP carboxyl terminus), Cyt b5 (cytochrome b5-like region), M-H (mind-bomb/HERC2 domain), CPH (conserved domain within Cullin 7, Parkin-like cytoplasmic -PARC- and HERC2), ZZ (ZZ-type zinc finger), DOC (domain homologous to subunit 10 of the anaphase promoting complex -APC)^10^

 The HERC protein family is homologous to E6AP C-terminus (HECT) E3 ubiquitin ligases three subfamilies, This HERC family consists of two subgroups based on size and domain architecture. The smaller proteins (HERC3–6) possess primarily HECT domain and one or more RCC1-like domains (RLDs), while the larger *HERC1* and *HERC2* proteins exhibit additional functional domains, including two RLDs, a C-terminal HECT, a WD40, a SPRY (spl A and RyR) domain, and several other minor motifs (Figure 2).^1,10^ Two characteristic domains: HECT and RLDs are essential in a number of important cellular processes such as cell cycle, cell signaling, and membrane trafficking.

 Extensive evidence indicates that mutations of the *HERC1* gene in humans lead to clinical syndromes altering key neurodevelopmental events with or without cerebellar affectation, resulting in intellectual disability and various neurological disorders such as epileptic seizures, dementia, and, in some cases, conditions related to the autism spectrum.^3,11,12^ The shared features seen in affected individuals with biallelic mutations in the *HERC1* gene led to the identification of the autosomal recessive neurodevelopmental disorder macrocephaly, dysmorphic facies, and psychomotor retardation (MDFPMR) syndrome (OMIM # 617011). Individuals affected by this condition exhibit distinct dysmorphic facial features and a consistently enlarged head; however, the initially increased birth weight tends to normalize as they age. Alongside these characteristics, additional neurological symptoms such as seizures, hypotonia, and gait ataxia may manifest. The overall presentation is marked by severe intellectual impairment in affected patients.^3,11-14^ Notably, a study by Pedrazza et al using Herc1-knockout mice, demonstrated the role of *HERC1* in regulation of osteoblastogenesis and osteoclastogenesis, influencing gene expression during mesenchymal stem cell differentiation. According to the article, *HERC1* deficiency leads to imbalanced bone homeostasis, causing osteopenia, with distinct effects in young female mice.^15^ The observed features in previously reported affected individuals, such as macrocephaly, dysmorphic facies, prominent forehead, long fingers, and vertebral column abnormalities, may be attributed to altered bone homeostasis.^15^

 Our patient presented with developmental delay, seizures, macrocephaly, hypotonia, unilateral hypertrophy, thoracic scoliosis, chest deformity, elevated homocysteine levels which normalized after treatment, and a normal brain MRI result (at the age of 6 months). Common features align with reported probands carrying *HERC1* mutations associated with MDFPMR syndrome, most of which are frameshift mutations leading to truncated *HERC1* proteins and loss of function. A comparative summary in Table 1 outlines these phenotypic similarities. Notably, our patient’s normal brain MRI result may be attributed to his young age at the time of assessment (6 months), as Ortega-Recalde et al in a case report presented initially normal MRI assessments in young siblings who later manifested communicating hydrocephalus, megalencephaly, and ventriculomegaly. However, normal brain MRI has been previously reported in another patient with *HERC1* loss-of-function mutations (see Table 1).^3,11-13^

**Table 1:** Comparing Clinical Characteristics in Patients with Reported *HERC1* Mutations, Including Our Case

		**Ortega-Recalde** **et al [2015]** ^3^		**Nguyen** **et al [2016]** ^11^	**Aggarwal et al [2016]** ^12^		**Utine et al [2017]** ^13^	**Presented case**
		**Patient 1**	**Patient 2**	**Proband **	**Patient 1**	**Patient 2**	**Proband **	**Proband**
Patient characteristics	Age	29 years	24 years	18 years	7 years	3 years	8 years	2 years
	Sex	Male	Female	Male	Male	Female	Male	Male
	HC at birth	NA	NA	37 cm ( + 2SD)	NA	Macrocephaly and ventriculomegaly on antenatal USG	NA	35 cm
	Birth weight	4 kg ( + 2SD)	NA	4 kg ( + 2SD)	4.5 kg ( + 3SD)	3 kg (-1SD)	4.9 kg ( + 3SD)	4 Kg ( + 2SD)
	Birth length	54 cm ( + 2SD)	NA	53 cm ( + 2SD)	NA	NA	NA	52 cm ( + 2SD)
Hypotonia		Yes	Yes	Yes	Yes	Yes	Yes	Yes
Spine		Kypho scoliosis	Kyphoscoliosis, Lumbar hyperlordosis	Normal	Mild kyphoscoliosis	Gibbus	Kyphoscoliosis	Thoracic scoliosis
Developmental delay		Yes	Yes	Yes	Yes	Yes	Yes	Yes
Facial characteristics	Prominent forehead	Yes	Yes	Yes	Yes	Yes	Yes	Yes
	Slant of palpebral fissures	Downslant	Downslant	NA	Upslant	Upslant	No	Downslant
	Proptosis	No	No	NA	Yes	Yes	No	?
	Hypertelorism	Yes	Yes	NA	Yes	Yes	No	Yes
	Long face	Yes	Yes	Yes	Yes	Yes	Slightly	Yes
	Ears	Normal	Normal	NA	Low set, large, posteriorly rotated	Low set, large, posteriorly rotated	Large	Low set, large
Neuroimaging		Normal	Communicating hydrocephalus, megalencephaly, ventriculomegaly	Megalencephaly, thick corpus callosum, small cerebellum	Shallow orbits, Metopic synostosis	NA	Normal	Normal
Seizures		Yes	NA	Yes	No	No	Abnormal EEG	Yes
Hypertrophy		NA	NA	NA	NA	Gum hypertrophy	NA	Yes

HC: head circumference, NA: Not available, SD: standard deviation.

 At the molecular level, the c.1280dup (p.4269Aspfs*33) variant found in our patient is considered likely pathogenic as it is predicted to create a premature stop codon potentially leading to the synthesis of a truncated 4300 residue-long protein. The mutant protein is predicted to lack the HECT domain located at the C-terminal region. However, it cannot be ruled out that the mRNA may become degraded by the nonsense-mediated decay (NMD) machinery, as seen in a study by Nguyen et al.^11^ In this study, the NM_003922.3:c.9748C > T (p.Arg3250*) variant in the *HERC1* gene resulted in complete absence of the *HERC1* protein in primary skin fibroblasts from the proband which suggest that the mentioned variant leads to NMD of *HERC1* mRNA.

 The loss of the HECT domain is likely of pathogenic significance as this domain interacts with the TSC1–TSC2 complex. This interaction suggests that *HERC1* functions as a crucial regulator in the mTOR pathway by acting as an ubiquitin ligase for TSC2, leading to its degradation and decreased stability. It has been indicated that TSC1, through complex formation with TSC2, prevents the association between TSC2 and the *HERC1* ubiquitin ligase.^2^

 The regulatory role of *HERC1* on mTOR pathway has also been shown in a functional study by Schwarz et al examining two siblings from a consanguineous marriage. These siblings were identified with a novel homozygous gain of function missense variant located in the C-terminal HECT domain of *HERC1* which results in mTORC1 hyperactivation, high phosphorylation of S6K1-kinase, and reduced autophagy during cell catabolism.^14^ The role of the mTOR pathway in the development and function of the brain has been supported in previous studies,^16^ which might explain the neurological findings in our patient.

 Considering the possibility of the NMD process due to the frameshift mutation found in our patient, the outcome would be the complete absence of *HERC1* mRNA. This absence, in turn, impacts other important domains like RLDs in *HERC1*. These RLD domains function as guanine nucleotide exchange factor (GEF) transporters for small G proteins, which bind to clathrin, associate with phosphatidylinositol 4–5 bisphosphate, and co-localize with actin polymers at membrane blebs,^1^ and underscores the role of *HERC1* in intracellular membrane trafficking. Loss of *HERC1* function might result in impaired protein trafficking and turnover. Since clathrin-mediated endocytosis mediates synaptic vesicle recycling,^17^ alterations to the normal clathrin cycle could disrupt normal synaptic function. Neuromuscular junction, peripheral nerve myelin, and various brain regions involved in learning processes are affected, highlighting the crucial role of *HERC1* in synaptic activity regulation during learning.^18^

 The novelty of the c.1280dup (p.4269Aspfs*33) variant within the *HERC1* gene amplifies its clinical relevance and highlights the need for continued expansion and refinement of genomic databases to catalog and characterize rare variants comprehensively. Further functional studies, such as *in-vitro* or animal models, are essential to elucidate the specific molecular consequences of this variant on protein structure, function, and downstream cellular pathways for establishing a direct link between the variant and the observed clinical phenotype.

 On the other hand, the CNV analysis revealed the heterozygous exon 1-5 duplication in the *PMP22* gene in our patient. This unique finding of the concomitant presence of a loss-of-function mutation in *HERC1* and a *PMP22* duplication holds significant importance, as it has not been documented in the existing literature. While this exact large *PMP22* duplication has not been reported before, *PMP22* duplications are commonly linked to peripheral neuropathies, such as CMT1A disease. *PMP22* is a large gene located in a 1.4 Mb region in chromosome17p.12. This region is prone to frequent genomic rearrangements.^9^ *PMP22* duplications leading to CMT1A are the most common form of *PMP22*-related neuropathy. These duplications usually present in the first two decades of life with difficulty walking or running. They are characterized by distal symmetrical muscle weakness and wasting, and sensory loss, with the legs more frequently and more severely affected than the arms.^19^ In a study, conducted on a Brazilian cohort of 53 families with demyelinating CMT, the disease onset was in the first decade in the majority of cases, including seven patients displaying abnormality since birth. Predominant initial symptoms included walking abnormalities and/or foot deformities and/or poor performance during children’s games (35/57). Less common onset manifestations comprised floppy baby syndrome, pain and/or cramps, hypoesthesia, hand weakness and/or deformity, severe scoliosis, and congenital hip dysplasia.^20^ According to the article, the onset of symptoms in individuals with *PMP22* duplication can vary greatly. Some individuals may present with symptoms at birth, while others may remain asymptomatic until adolescence or beyond. While our patient’s central nervous system involvement and musculoskeletal anomalies deviate from the classical manifestations of *PMP22*-related disorders, the coexistence of the homozygous *HERC1* variant and the heterozygous *PMP22* duplication raises intriguing questions regarding potential synergistic or modifying effects contributing to the observed phenotype such as scoliosis.

 Interestingly, researchers found that the *HERC1* protein may be important for the production of myelin. In a study by Bachiller et al on the tambaleante (tbl) mouse, a Gly483Glu mutant mouse that exhibits cerebellar ataxia and Purkinje cell death due to extensive autophagy, they found that experiencing reduced vesicle availability at the neuromuscular junction before cerebellar degeneration, exhibits altered motor function. Functional analysis revealed delayed action potential propagation and morphological damage in glial cells, including tomacula and hypermyelination. Non-myelinated terminal Schwann cells at the neuromuscular junction were also altered, and phosphorylated Akt-2 increased in the sciatic nerve. Proposing a molecular model, they suggested how the mutated *HERC1* in tbl mice affects peripheral nervous system myelination.^21^ Therefore, simultaneous occurrence of *HERC1* loss-of-function mutation and *PMP22* duplication in our patient suggests potential synergistic effects. Abnormalities in myelin patterns and thicker myelin layers observed in the mouse model emphasizes the complex interplay between *HERC1* and *PMP22*, potentially influencing peripheral nervous system myelination and contributing to motor dysfunction.

## Conclusion

 In conclusion, identification of the novel homozygous likely pathogenic variant in *HERC1* (c.1280dup, p.Ile469Aspfs*33) in this case underscores its significance as a potential driver of the complex clinical presentation observed in our patient. Our finding expands the allelic spectrum of the *HERC1* gene mutations, and emphasizes the importance of CNV analysis along with SNV and small indel investigations for early detection and diagnosis of concomitant pathogenic CNVs such as *PMP22* duplication. Importantly, the co-occurrence of a novel loss-of-function mutation in *HERC1* and a *PMP22* duplication, an unprecedented finding in the literature, adds a unique layer to our understanding of genetic complexity in pediatric cases. In our case, early intervention can potentially slow the progression of the disease and improve the quality of life for affected individuals. This case contributes to expanding the knowledge base of rare genetic variants and their implications in complex pediatric presentations, ultimately paving the way for more precise diagnostic and therapeutic strategies in such cases.

## Supplementary Files


Supplementary file. The Final Candidate Variants.
